# Diet in Inflammatory Bowel Diseases: Efficacy, Tolerability, and Microbiome Effects Toward Personalized Management

**DOI:** 10.1007/s10620-025-09590-y

**Published:** 2025-11-28

**Authors:** Francesco Calabrese, Andrea Pasta, Elena Formisano, Elisa Marabotto, Manuele Furnari, Livia Pisciotta, Patrizia Zentilin, Edoardo G. Giannini, Giorgia Bodini

**Affiliations:** 1 Gastroenterology Unit, Department of Internal Medicine, University of Genoa, IRCCS‐Ospedale Policlinico San Martino, Viale Benedetto XV, 6, 16132 Genoa, Italy; 2 Gastroenterology Unit, Department of Internal Medicine, University of Genoa, 16132 Genoa, Italy; 3 Dietetics and Clinical Nutrition Unit, Department of Internal Medicine, University of Genoa, 16132 Genoa, Liguria Italy

**Keywords:** Ulcerative colitis, Crohn’s disease, Exclusion diet, Low-FODMAP diet, Gut microbiota

## Abstract

**Introduction:**

Inflammatory bowel disease (IBD) is increasingly recognized as a condition in which diet is not only a trigger for symptoms but also a potential tool for disease modulation. Mounting evidence links dietary patterns and specific nutrients to intestinal inflammation, microbiome composition, and mucosal repair, opening new avenues for personalized therapy.

**Diet interventions:**

Exclusive and partial enteral nutrition remain among the most effective nonpharmacologic strategies in Crohn’s disease, while emerging data support the Mediterranean diet, specific carbohydrate diet, and low Fermentable Oligo-, Di-, Mono-saccharides And Polyols (FODMAP) approaches for symptom relief and quality-of-life improvement.

**Gaps and future directions:**

Nevertheless, evidence is often heterogeneous, with variability in dietary protocols, endpoints, and patient populations, making firm recommendations challenging. Adherence and long-term sustainability remain major barriers, underscoring the need for realistic, patient-centered approaches. By integrating current findings with clinical practice, diet can evolve from supportive care to a central component of multidisciplinary IBD management—if guided by robust evidence and tailored to individual needs. As research continues to refine the role of specific foods, nutrients, and patterns, dietary therapy holds promise not only to complement pharmacologic treatments but also to address the broader nutritional and metabolic needs of patients living with IBD.

## Introduction

Crohn’s disease (CD) and ulcerative colitis (UC) are chronic inflammatory bowel diseases characterized by relapsing–remitting gastrointestinal inflammation [1–3]. The etiology involves a complex interplay of genetic susceptibility, immune dysregulation, gut microbiome alterations, and environmental factors such as diet, smoking, and antibiotic use [4–6]. Notably, the global incidence of IBD has risen in regions adopting a Westernized diet high in fats, refined sugars, and processed foods and low in fiber [7, 8]. This epidemiological shift implicates diet as both a potential contributor to IBD pathogenesis and a promising modifiable factor in disease management [8]. Indeed, diet influences gut inflammation by altering the intestinal barrier, immune responses, and the composition and function of the gut microbiota, including in the post-surgical setting [9–11].

Despite advances in medical therapies, many patients fail to achieve sustained remission or experience drug side effects [12–14]. Dietary therapy offers an attractive, non-pharmacological approach to complement standard treatments, particularly in the pediatric setting, where a major concern with immunosuppressants is long-term safety [15, 16]. Exclusive enteral nutrition (EEN) has become an established induction therapy for pediatric CD, highlighting that diet can have disease-modifying effects [15, 17]. However, in adult practice, the integration of diet into IBD management is still evolving, and dietary interventions are gaining popularity [15, 18]. Therefore, we systematically evaluate the role, efficacy, and patient acceptance of various diets in both CD and UC.

**Fig. 1:**
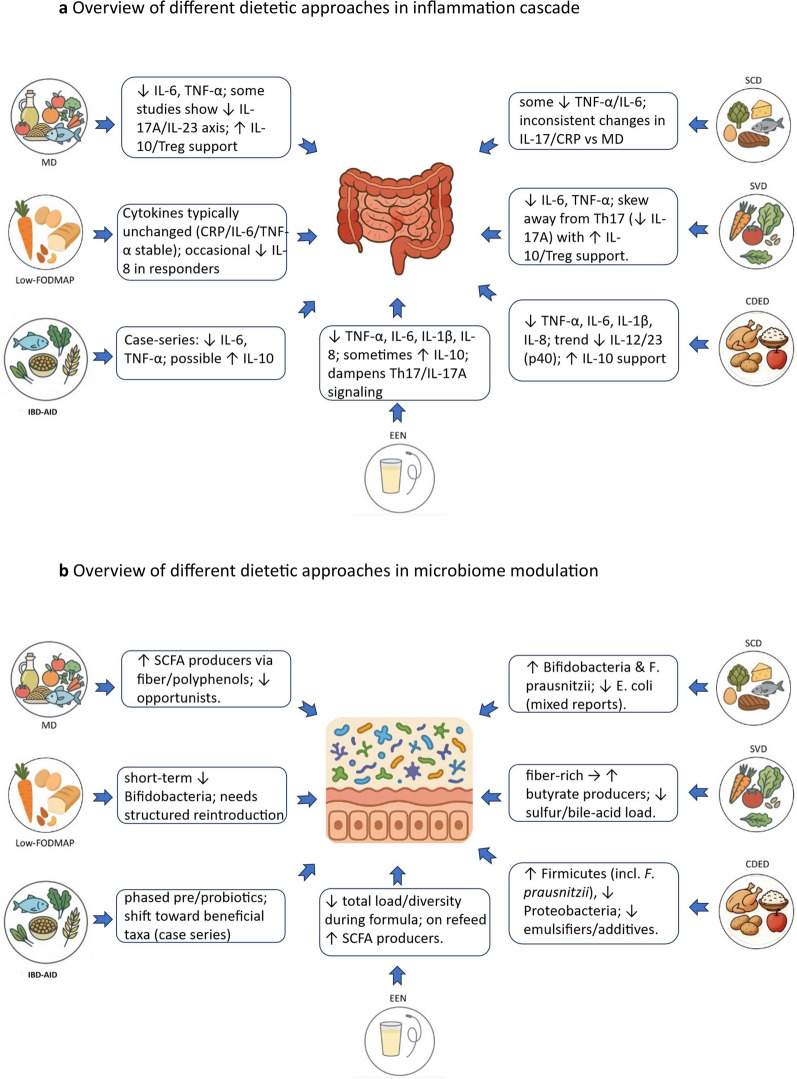
Overview of different dietetic approaches in inflammation cascade, **b** Overview of different dietetic approaches in microbiome modulation

Given these considerations, this review analyzes the current literature on major dietary interventions in IBD, encompassing both adult and pediatric studies in CD and UC (Fig. 1). For each dietary strategy, we provided a summary of its clinical efficacy (in both induction and maintenance of remission), proposed mechanisms of action, tolerance and adherence data, and its impact on the gut microbiome and inflammatory markers. By organizing diets by strength of evidence, we aim to clarify which nutritional approaches are most supported and how they can be implemented. Ultimately, this review seeks to inform a more personalized, evidence-based use of diet as a cornerstone of holistic IBD care.

## Methods

We conducted a narrative critical review of the literature, searching in databases (PubMed and Embase) for studies published up to 2025 examining dietary interventions in CD or UC. Search terms included combinations of *IBD, CD, colitis* with specific diets (e.g., *enteral nutrition, exclusion diet, specific carbohydrate diet, Mediterranean, low-FODMAP, semi-vegetarian, IBD-AID*) and related keywords. We included randomized controlled trials (RCTs), cohort and case–control studies, systematic reviews/meta-analyses, and significant case series that reported on clinical outcomes (remission rates, symptom scores, biomarker changes), adherence, or microbiome/inflammatory impacts of diets in IBD. Both adult and pediatric populations were included. We excluded single-patient case reports and studies focusing only on dietary risk factors for IBD (rather than interventions).

Data were extracted on study design, sample size, IBD type, population (age), diet intervention details, comparator (if any), outcomes (clinical remission, response, endoscopic healing, etc.), adherence measures, and key findings on microbiota or inflammatory markers. We synthesized results by diet type, and for each diet we summarized the level of evidence (e.g., established therapy, emerging evidence, or limited data).

A summary table (Table 1) is provided to compare key outcomes and considerations across diets.

**Table 1:** Comparative Summary of Dietary Interventions in IBD

Diet (intervention)	Efficacy (clinical outcomes)	Adherence/tolerance	Key limitations	Population studied
Exclusive Enteral Nutrition (EEN)	Induces remission in 60–80% of pediatric CD’s (comparable to steroids). Improves mucosal healing (89% vs 17% vs steroids in one trial) [19] In adults, lower efficacy (45% remission vs 73% steroids) [39] Minimal effect in UC (198)	Poor long-term adherence in adults; moderate in children. Monotonous all-liquid diet – challenges with palatability and social acceptance. Often requires NG tube in kids	Complete food exclusion (difficult beyond 6–8 weeks). Risk of compliance failure and weight loss if not meeting calorie needs. Not a maintenance strategy (must transition to food)	Primarily pediatric CD’s disease (first-line induction) [15]. Some trials in adult CD’s (adjunctive). Not routinely used in ulcerative colitis
CD’s Disease Exclusion Diet (CDED) + Partial EN	High induction remission in mild-mod CD: *Adults*: 6-week clinical remission 62.5%; among responders, 80% maintained remission at 24 weeks; 35% endoscopic remission at 24 weeks *Pediatrics*: 12-week corticosteroid-free remission 75.6% (CDED + PEN) vs 45.1% (EEN)[22]. Sustains remission with ongoing diet (80% of initial responders remained in remission at 24 weeks in adults) [69]. Shown to reduce CRP & calprotectin, and some endoscopic healing. No RCT data in UC	Good adherence relative to EEN – allows some normal food (97% adherence at 6 weeks in kids) [22]. Patients find it more palatable; still requires commitment to specific food list. Tolerance good; some may tire of limited choices over time	Requires access to specific allowed foods and formula. Moderately restrictive (gluten, dairy, many processed foods excluded). Needs dietitian coaching. Long-term monotony can occur	CD’s disease – studied in children and adults (mild to moderate). Not tested in UC (concept being adapted in trials for UC)
Specific Carbohydrate Diet (SCD)	Anecdotal and case-series evidence of symptom relief and reduced markers in some CD & UC patients. RCT in CD’s: not superior to Mediterranean diet for remission (46% vs 43% at 6 weeks) [88]. Can improve stool frequency, pain in some; few data on mucosal healing (mostly none)	Difficult to adhere long-term (eliminates all grains, most dairy, sugars). Requires cooking from scratch. Those who see improvement often stick with it; others lapse due to cravings or social limits. Tolerance: generally safe, but risk of weight loss if intake insufficient	Very restrictive – risk of nutritional deficiencies (folate, B-vits, calcium) without supplementation. Lack of robust evidence for inflammation control. Hard to follow in social situations; limited restaurant options	Used by both pediatric and adult patients with IBD (CD’s > UC) in case reports. RCT evidence in adults with CD’s (199). Pediatric use mostly in CD’s (small cohorts)
Mediterranean Diet (MD)	Improves overall health; in CD’s, matched SCD in RCT for symptom remission (199). Associated with lower inflammatory markers and possibly fewer flares (observational). No direct evidence of induction of remission in active flares, but likely beneficial for maintenance	Excellent palatability and high adherence potential. Wide variety of foods, flexible guidelines. Patients enjoy foods (olive oil, fish, fruits). Easy to maintain long-term as a lifestyle diet	May not be sufficient as sole induction therapy for moderate/severe IBD. Relies on patient making healthy choices – some may overdo “allowed” items (like wine). Benefits are modest if disease is aggressive (needs meds too)	Studied mostly in adult CD’s (e.g., DINE-CD trial) (199). Recommended broadly for IBD adults for overall health. Limited specific research in pediatric IBD or UC (though likely applicable)
Low-FODMAP Diet	Effective for relieving functional GI symptoms in IBD (bloating, diarrhea in IBS-like patients) [125]. 50–60% report symptom improvement vs 15% on control diet [125]. Not intended to reduce IBD inflammation; clinical disease indices typically unchanged (in remission patients)	Short-term adherence good (4–6 week elimination) with dietitian support. Symptom relief reinforces compliance. Reintroduction phase allows personalization, improving long-term tolerance. Some find diet complex to learn, but many can partially adopt it indefinitely	Does not address inflammation – purely symptomatic. Strict phase is nutritionally restrictive (low fiber, low-FODMAP can reduce Bifidobacteria). Must reintroduce foods to avoid long-term microbiota impact and nutrient gaps. Requires patient education for proper execution	Adults with IBD in remission or mild disease who have IBS-type symptoms (studied in both CD’s and UC) [125]. Some use in pediatric IBS, but not widely studied in pediatric IBD
Semi-Vegetarian/Plant-Based Diet	Very promising maintenance results in CD’s: 2-year remission 92% on semi-vegetarian diet vs 33% on regular diet in one study [154]. Associated with fewer relapses (small cohort) and improved well-being. Lacks RCT validation. In UC, no specific trials; plant-based diets likely beneficial but evidence mostly extrapolated	Moderately high adherence if motivated – diet is less restrictive than vegan (allows occasional fish/meat). 73% adhered at 2 years in CD’s study [154]. Diet is abundant and satiating with proper planning. Socially acceptable (many vegetarian options in restaurants)	Limited evidence base. Some patients may struggle without meat daily if culturally accustomed. Must ensure adequate protein, B12, iron from plant sources. Changes need to be gradual to avoid GI bloating from sudden fiber increase	CD’s disease – adult maintenance phase (post-remission) [154]. Single-center data from Japan. Also general recommendation for patients with IBD interested in nutrition. No dedicated pediatric data, though generally safe in older kids with guidance
IBD Anti-Inflammatory Diet (IBD-AID)	Case series show 60% achieved good clinical response [161]. All patients who adhered had symptom improvement; some went off medications [161]. Not yet proven in RCT. Appears to reduce diarrhea and CRP in anecdotal reports. Both CD’s and UC patients have benefited in reports	Initial barriers (33% refused to start in series) [161], but among adopters, decent adherence. More flexible than SCD: allows gradual reintroduction of grains, etc., which improves sustainability. Many find it manageable long-term with dietitian help, as food variety increases in later phases	Evidence mainly low-quality (no control group). Requires meal prep and education on phases. Still excludes gluten and refined foods – can be challenging for some. Without commitment, partial adherence may yield less benefit (though partial adherence still possibly helpful)	Both CD’s and UC (adult patients, 19–70 yrs) [161]. Implemented in some centers as adjunct therapy. No formal pediatric studies, but diet principles used in teens (with modifications)

*EEN* Exclusive Enteral Nutrition, *CDED* CD’s Disease Exclusion Diet, *SCD* Specific Carbohydrate Diet, *FODMAP* Fermentable Oligo-, Di-, Monosaccharides and Polyols, *IBD-AID* IBD Anti-Inflammatory Diet. “Remission” refers to clinical remission (symptom-based, with or without biomarker normalization) unless specified as endoscopic remission. Adherence ratings are relative and assume patients have access to dietitian guidance. Each diet’s success can vary greatly between individuals

## Diet Interventions

Multiple dietary strategies have been investigated in IBD, each with differing levels of evidence and unique practical considerations. Importantly, dietary approaches serve two distinct purposes: some are *therapeutic* and aim to modify intestinal inflammation and induce or maintain remission (e.g., exclusive enteral nutrition, Crohn disease exclusion diet, certain whole-food patterns), whereas others are primarily *symptom-directed* and target irritable bowel syndrome (IBS)-type functional symptoms in patients who are already in inflammatory remission (most notably the low-FODMAP diet). Broadly, diets can be categorized as: [1] Formula-based therapies (liquid enteral nutrition), [2] Exclusion diets that eliminate specific food components, [3] Whole-food patterns emphasizing certain food groups, and [4] Symptom-directed diets. Table 1 provides a comparative summary of these diets, including their efficacy, adherence, limitations, and the populations in which they have been studied.

## Exclusive Enteral Nutrition (EEN)

EEN entails using a nutritionally complete liquid formula as the sole source of nutrition for a defined period (typically 6–8 weeks) while excluding all normal foods [19–21]. Formulas may be elemental or polymeric. EEN is most commonly used in CD, where it induces remission particularly in children [19, 22].

Numerous studies and meta-analyses have shown that in children with active CD, EEN induces clinical remission in roughly 60–80% of cases, comparable to corticosteroid therapy [19, 23]. A meta-analysis of pediatric trials found no significant difference in remission rates between EEN and steroids, confirming that EEN is as efficacious as corticosteroids for induction of remission – but without steroid-related side effects [23, 24]. Importantly, EEN may achieve deeper healing: one prospective trial in children reported significantly higher mucosal healing rates at 8 weeks with EEN (89%) versus steroids (17%) [19]. Consequently, EEN is recommended as first-line induction therapy in pediatric CD, as reaffirmed by the 2025 ECCO Dietary Management Consensus. [18, 25].

In adults with CD’s, EEN is less commonly used due to tolerance issues, but smaller studies suggest it can induce remission in roughly 15–50% of adults, often as an adjunct to medical therapy [26, 27]. EEN has not been established as a primary therapy for UC – clinical response in UC is modest at best, and EEN is not routinely utilized for patients with UC [28].

EEN is believed to exert its effects by promoting 'bowel rest' and reducing exposure to dietary antigens that may trigger mucosal inflammation [29]. By providing an exclusively absorbable formula, EEN minimizes exposure to whole-food components such as certain proteins, fats, and additives that may exacerbate inflammation [30, 31]. EEN leads to a marked (if paradoxical) reduction in gut microbial diversity during treatment [32]. This may “starve out” pathogenic bacteria and alter the microbiome to a less pro-inflammatory composition once refeeding occurs [26, 32]. EEN has been shown to directly reduce mucosal pro-inflammatory cytokine levels (e.g., TNFα, IL-1β, IL-6) and to improve intestinal barrier function [33, 34]. Enhanced mucosal healing with EEN, compared to steroids, suggests that the diet not only reduces inflammation but also allows the gut lining to repair [19, 35]. Improved nutritional status and provision of key nutrients (e.g., glutamine, arginine) may further support immunoregulation and barrier integrity [36, 37].

### Tolerance and Adherence

Adherence to EEN can be challenging, particularly for adolescents and adults, due to both practical and sensory barriers. The regimen typically requires patients to consume only a liquid formula—either orally or via a nasogastric tube—with complete exclusion of normal food. This dietary restriction is often perceived as socially isolating and difficult to maintain in daily life, especially in adults who report issues such as poor palatability, monotony, and taste fatigue [38, 39]. The use of elemental formulas, which contain free amino acids and have a characteristically bitter taste and low fat content, has been frequently associated with poor adherence, especially when administered orally [40, 41]. In contrast, polymeric formulas, which contain whole proteins and a higher fat content, are generally more palatable and better tolerated [42, 43]. In pediatric populations, EEN adherence is often moderate to good, particularly when supported by motivated families and experienced dietitians. For example, one center reported that up to 74% of children completed a full course of EEN induction therapy [44]. Pediatric studies using polymeric formulas like Modulen IBD® have shown high adherence rates when the formula is administered orally, with nasogastric tube support used selectively to help meet nutritional targets [45, 46]. Interestingly, poor adherence in children is often more related to the required formula volume rather than taste, suggesting that concentrated formulas (e.g., 1.5 kcal/mL) could improve tolerability in both pediatric and adult cohorts [45–47]. In adults, however, adherence remains significantly lower– monotony of formula and difficulty abstaining from solid food lead to high dropout rates [43]. Studies report higher dropout rates in adults using elemental or semi-elemental diets compared to those receiving polymeric formulas SPS:refid::bib39[39]. Side effects like taste fatigue, nausea, constipation or refeeding syndrome can occur [48, 49]. Because long-term EEN is impractical, it is usually given for 6–8 weeks to induce remission, then patients transition to either a less restrictive diet or maintenance medications [50].

Most mechanistic and biomarker evidence derives from pediatric cohorts; adult data remain more limited.

### Impact on Microbiota and Inflammation

EEN causes notable shifts in the gut microbiota. During EEN, overall bacterial load and diversity decrease significantly [34]. Some beneficial fiber-fermenting species decline due to lack of fiber intake [51]. Interestingly, EEN-induced remission is associated with an increase in certain bacterial groups upon reintroduction of foods – one study noted enrichment of *Ruminococcaceae* (SCFA-producing bacteria) in patients achieving mucosal healing with EEN [19, 52]. The reduction in microbial diversity during EEN, while counterintuitive (since higher diversity is usually healthier), may help suppress pathogenic taxa and their fermentation products that drive inflammation [53]. In terms of inflammatory markers, EEN consistently reduces C-reactive protein (CRP) and fecal calprotectin in responding patients, often more effectively than steroids [19]. EEN’s exclusive nature also eliminates dietary emulsifiers, saturated fats, and other additives that have been shown to increase intestinal permeability and endotoxin release [54]. Overall, EEN’s profound impact on both inflammation and microbiota underpins its therapeutic benefit in CD.

### Mechanistic Paradox: Processed Composition of EEN Formulas

Compositional analyses of effective EEN formulas reveal a profile that appears at odds with common mechanistic theories of dietary benefit in IBD. Most products are animal/dairy-protein based, rely heavily on maltodextrin, and contain multiple food additives and emulsifiers, including modified starches, carrageenan, carboxymethyl cellulose, and polysorbate-80 [55]. Despite this, remission rates are similar between formulas with and without these additives, even when restricting analyses to RCT-tested products. These findings challenge the assumption that therapeutic benefit necessarily derives from avoiding animal protein or ultra-processed components, and suggest that EEN’s efficacy likely reflects broader shifts in nutrient delivery, antigen exclusion, and microbial/metabolic recalibration, rather than elimination of specific “harmful” ingredients.

## Partial Enteral Nutrition

Given the difficulty of strict EEN, partial enteral nutrition (PEN) has been explored [56]. PEN involves providing a substantial proportion of calories from formula while allowing some limited food intake [56, 57]. By easing the psychological burden of zero food, PEN can improve adherence [57]. However, PEN alone is generally less effective for inducing remission than full EEN [56, 57]. Clinical interest has shifted to combining PEN with specific elimination diets (notably the Crohn Disease Exclusion Diet) to balance efficacy and tolerability [58]. For example, providing 50% of calories from formula and the rest from a defined whole-food diet has shown success in pediatric trials [59]. However, adult evidence is weaker.

## Crohn Disease Exclusion Diet (CDED)

The CDED is a whole-food diet specifically designed for CD, aimed at excluding food components thought to exacerbate gut inflammation and dysbiosis [60]. The CDED involves two phases with a defined list of allowed foods [60]. In general, CDED restricts dairy, gluten, animal fats, red and processed meats, and artificial additives/emulsifiers, while emphasizing easily digestible foods, select carbohydrates, lean proteins, and fruits/vegetables that are lower in insoluble fiber [61, 62].

The CDED is an emerging dietary therapy with growing evidence, particularly in mild-to-moderate CD [63]. A landmark randomized controlled trial in children compared 12 weeks of CDED + PEN vs. standard EEN [64]. At week 6, remission rates were similar, but by week 12 the CDED + PEN arm had significantly more children in corticosteroid-free remission (75.6% vs 45.1% with EEN followed by reintroduction; *P* = 0.01) [64]. This demonstrated that a partial diet can maintain remission beyond the initial induction period better than returning to an unrestricted diet after EEN.

In adults, a recent open-label trial (CDED-AD) evaluated CDED with or without PEN in biologic-naïve adults with mild-to-moderate CD’s [58]. After 6 weeks, 62.5% achieved clinical remission; among these responders, 80% sustained remission through 24 weeks on diet therapy [58]. By week 24, 35% of the 40 patients had achieved endoscopic remission as well [58]. These results in adults, though from a pilot study, underscore the diet’s potential for both induction and maintenance of remission. In a recent open-label randomized controlled trial, CDED without PEN was superior to a Mediterranean diet in inducing and maintaining clinical remission at both 12 and 24 weeks in adults with mild-to-moderate CD, with significant improvements in inflammatory markers and favorable changes in body composition, including reductions in fat mass and preservation of lean body mass [62]. Notably, CDED has not been formally studied in UC; it is tailored to CD’s mechanisms. An exclusion diet for UC, known as the Ulcerative Colitis Exclusion Diet (UCED), was evaluated in a RCT involving patients with active, medication-refractory UC, comparing UCED alone, fecal transplantation (FT), and FT combined with UCED; the diet alone achieved the highest rates of steroid-free clinical remission at 8 weeks (40%), endoscopic remission (27%), and was the only intervention to induce mucosal healing (20%), outperforming FT with or without dietary conditioning, although most differences did not reach statistical significance [65].

The CDED was formulated based on the hypothesis that certain dietary components adversely affect the gut microbiome and intestinal barrier in CD’s [60, 66]. By excluding pro-inflammatory foods (such as ultra-processed items high in emulsifiers, maltodextrin, and sulfites, as well as gluten and high-lactose dairy) the diet aims to remove microbial and molecular triggers of inflammation [61, 66]. At the same time, the allowed foods provide key nutrients and prebiotics to support beneficial bacteria and mucosal healing [63, 67]. Mechanistically, CDED + PEN has been shown to increase microbial diversity after the initial phase and promote growth of bacteria associated with a healthy mucosa (like *Firmicutes*) while reducing *Proteobacteria* that often bloom in CD’s flares [58]. The inclusion of resistant starch and fibers from fruits, vegetables, and oats (in later phases) fosters short-chain fatty acid production (butyrate, propionate), which has anti-inflammatory effects on colonic cells [66, 68]. In essence, CDED modulates the microbiome by depriving pro-inflammatory bacteria of their fuel (e.g., certain fats, polysaccharides, additives) and encouraging a milieu that favors anti-inflammatory bacteria and metabolites [22]. The diet also tends to be low in red meat and high in specific fish or plant proteins, thus lowering sulfur-rich amino acids that can produce harmful luminal metabolites [66]. Together, these changes lead to reduced inflammatory activity; indeed, patients on CDED have shown significant decreases in fecal calprotectin and CRP during treatment in the trials [22, 62, 69].

### Tolerance and Adherence

One of CDED’s strengths is improved tolerability relative to EEN [64]. Patients are allowed to eat a variety of normal-texture foods (though limited in choice), which helps with satiety, taste satisfaction, and social acceptance. As noted, pediatric patients significantly preferred CDED over strict EEN in trials [64]. In practice, adherence to CDED still requires motivation and education, as the diet is restrictive and phased (certain foods are only reintroduced after the initial 6-week phase). Moreover, adherence was better in the CDED group – 97.5% of children managed to follow CDED for 12 weeks, versus 73.6% tolerating EEN for 6 weeks in that study [64]. Over the longer term (12–24 weeks), adherence rates may decline due to dietary monotony and practical limitations related to dining outside the home. Frequently reported barriers include persistent cravings for excluded foods (e.g., sweets, bread), the necessity for detailed meal planning, and the financial or logistical burden associated with sourcing specialized dietary products. However, allowing *some* regular food (versus none in EEN) greatly improves willingness to continue the therapy. Digestive tolerance of the diet is generally good; initial mild constipation can occur due to low fiber in the very early phase, but this improves as fruits/vegetables are increased later. Overall, CDED plus partial formula has emerged as a feasible alternative to EEN, balancing efficacy with patient acceptance, according also to ECCO consensus [18, 62].

### Impact on Microbiota and Inflammation

The CDED’s impact on the gut microbiome is a central aspect of its rationale. Studies have shown that patients on CDED experience an increase in beneficial microbial diversity as inflammation diminishes [58]. In the adult CDED-AD trial, both CDED + PEN and CDED-alone groups showed changes in microbiota composition by week 24, including increased representation of *Firmicutes* and reductions in *Proteobacteria*, although differences between the groups were not statistically significant [58]. Certain SCFA-producing taxa (like *Roseburia* and *Faecalibacterium prausnitzii*) tend to increase on CDED, which could contribute to mucosal healing [67, 68, 70]. From an inflammation standpoint, patients on CDED have demonstrated significant drops in fecal calprotectin levels (often into normal range when remission is achieved) and normalization of CRP in many cases [58, 62, 64]. The diet’s low content of emulsifiers and additives is believed to help restore gut barrier function, reducing endotoxin leakage that drives systemic inflammation [71]. Additionally, by restricting high-fat animal products, CDED may reduce pro-inflammatory molecules like secondary bile acids and sulfide produced by meat-metabolizing bacteria [66, 72]. In summary, the CDED may help in ameliorating microbiome’s composition and biochemical environment, resulting in lowered inflammatory burden in the gut. This diet exemplifies a targeted strategy to alter the food–microbiome–inflammation axis in CD.

## Specific Carbohydrate Diet (SCD)

The Specific Carbohydrate Diet is a nutritionally balanced but restrictive diet that eliminates most complex carbohydrates and all refined sugars and grains [73]. Originally developed for celiac and other gastrointestinal disorders, SCD permits monosaccharides (glucose, fructose) but excludes disaccharides (lactose, sucrose) and most polysaccharides (starches) [73, 74]. In practice, SCD allows fruits, non-starchy vegetables, meats, nuts, fermented yogurt, and certain legumes, but bans grains (wheat, corn, rice, etc.), potatoes, processed foods, dairy (except fermented yogurt or certain aged cheeses), and added sugars [73, 75]. The rationale is that “specific” easily digestible carbs are absorbed higher in the gut, starving pathogenic bacteria in the colon and reducing fermentation byproducts that can fuel inflammation.

Although the diet is widely promoted in patient communities, the scientific evidence supporting SCD in IBD remains limited and derives mostly from small, uncontrolled pediatric case series or culturally specific cohorts, which restricts generalizability. Several pediatric case series and cohort studies suggested that SCD can improve symptoms and inflammatory markers in IBD. A retrospective series reported that SCD or modified low-carb diets were associated with clinical improvement in seven children with CD’s [76]. A more recent pediatric RCT comparing three versions of the Specific Carbohydrate Diet (standard SCD, modified SCD, and a whole foods diet) found that all patients completing the 12-week intervention achieved clinical remission, with the more exclusionary diets leading to greater reductions in CRP and shifts in gut microbiota composition, highlighting a potential dose–response relationship between dietary restriction and inflammatory control [75]. Regarding adults population, in the DINE-CD trial, a randomized study of 194 adults with mild-to-moderate CD, the SCD was not superior to the Mediterranean diet (MD) in achieving symptomatic remission at 6 weeks (46.5% vs. 43.5%), nor in secondary outcomes such as fecal calprotectin or CRP response [77]. By 12 weeks, no significant differences were observed between groups. Given its comparable efficacy, greater ease of adherence, and broader health benefits, the authors concluded that the MD may be preferable for most patients with mild-to-moderate CD’s [77]. Outside of CD’s setting, SCD has also been tried in UC in small studies, but data are sparse. One small UC trial comparing SCD to a low-residue diet found similar symptomatic outcomes [78]. Overall, SCD cannot be considered an evidence-based primary therapy at this time, though individual patients may experience benefit, and ECCO consensus does not recommend due to insufficient evidence [18].

The hypothesis behind SCD is that poorly absorbed carbohydrates foster dysbiosis and intestinal injury. By eliminating lactose, sucrose, and starch, SCD reduces substrate for gas-producing and potentially pathogenic gut bacteria [73, 76, 79]. This in turn might decrease bacterial overgrowth and production of toxins or antigens that trigger inflammation [76]. SCD’s allowance of only simple sugars means carbs are absorbed in the upper small intestine, leaving little for colonic microbes – somewhat akin to the effect of EEN in “starving” the colon [66]. Some evidence supports that SCD can indeed alter the microbiome: one study of pediatric patients with IBD on SCD noted increased microbial diversity and shifts in functional pathways of gut bacteria, correlating with clinical improvement [67]. SCD also shares features with a paleolithic-style diet, being grain-free and additive-free, which might remove pro-inflammatory dietary components like gluten (in susceptible individuals) or emulsifiers [80]. The diet includes fermentable fiber from fruits, veggies, and nuts, so it’s not fiber-free; this fiber could promote beneficial SCFAs, though the lack of whole grains means certain fiber types (resistant starch) are missing [81]. Notably, SCD is quite high in protein and fat due to reliance on meat and nuts – this raises questions about long-term effects, as high animal fat can be pro-inflammatory [82]. The absence of refined sugar is a clear positive, as high sugar is linked to inflammation and dysbiosis [83]. In summary, SCD could act by modifying gut microbiota composition and metabolic outputs, cutting out known dietary offenders, and possibly inducing a caloric ketosis (due to low carbs) which might have anti-inflammatory effects. These mechanisms remain theoretical pending more rigorous study.

### Tolerance and Adherence

The SCD is among the more restrictive diets, which poses challenges for adherence. It requires eliminating staple foods like bread, pasta, rice, milk, and sugar – which can be a drastic lifestyle change. Patients must cook most meals from allowed ingredients; this can be time-consuming and socially limiting (e.g., dining out is difficult) [84]. Early enthusiasm can wane as the monotony and restrictiveness of the diet set in, resulting in a subset of patients which cannot maintain SCD long-term [84, 85]. Common drop-out reasons include weight loss (since the diet can be low in calories if not carefully planned), cravings for forbidden foods, and difficulty managing the diet during travel or social events [85]. Nutritional adequacy is a concern: without grains and many dairy products, patients risk deficiencies in B-vitamins, calcium, vitamin D, etc., unless compensated through other allowed foods or supplements. That said, a motivated group of patients does adhere strictly and report high satisfaction if their symptoms improve. In the DINE-CD trial, adherence to both SCD and MD was high during the 12-week study (likely due to intensive dietician support and close monitoring) [77]. Outside of trial settings, SCD adherents often rely on community support (online forums, SCD cookbooks) to sustain the diet. Tolerance-wise, SCD foods are generally well-tolerated (it’s a whole-food diet after all). Some patients experience initial GI upset as their microbiome adapts – e.g., increased fiber from fruits/nuts might cause bloating until the gut adjusts. Over longer term, if SCD leads to symptom control, patients often report feeling better energy and nutritional status; but if it doesn’t help within a few months, people tend to abandon it.

### Impact on Microbiota and Inflammation

SCD’s effects on the microbiome are actively being studied. An SCD-like diet seems to increase microbial diversity and altered the gut microbiome functional profile, consistent with a “healthier” community, alongside symptom improvement [66, 86]. Specifically, some patients on SCD have shown an increase in beneficial genera such as *Bifidobacteria* and *Faecalibacterium*, and a decrease in potential pathogens like *Escherichia coli *[66, 76]. This is likely due to removal of refined sugars that feed blooms of *Proteobacteria*, as well as the inclusion of yogurts providing probiotics (SCD encourages homemade fermented yogurt rich in *Lactobacillus* and *Bifidobacterium*) [83, 87]. In Suskind’s pediatric study, SCD led to reduced CRP and a downward trend in fecal calprotectin, though not all patients normalized these markers [75]. In contrast, the adult RCT by Lewis et al. found no significant differences between SCD and the MD in reducing CRP or calprotectin at 6 or 12 weeks, suggesting similar anti-inflammatory effects [88]. While SCD may help alleviate symptoms like bloating or diarrhea by limiting fermentable carbs, there’s no evidence it heals mucosa or alters long-term disease course [89]. Overall, SCD may modulate the microbiota and reduce some inflammatory outputs, but high-quality evidence of its disease-modifying effect in IBD is still lacking.

## Mediterranean Diet (MD)

The MD refers to a dietary pattern inspired by traditional eating habits of countries bordering the Mediterranean Sea. It is characterized by high intake of fruits, vegetables, legumes, nuts, and whole grains; use of olive oil as the primary fat; moderate consumption of fish and poultry; low intake of red meat, processed meats, and sweets; and often moderate wine consumption with meals [90]. The MD is not an exclusion diet per se, but rather an emphasis on plant-based, high-fiber, and unsaturated fat-rich foods with limited processed items. It is widely regarded as an anti-inflammatory and cardioprotective diet in the general population [91, 92].

The Mediterranean diet has been associated with numerous health benefits, and in context of IBD, it has shown promise as a maintenance diet or as a comparison diet in trials [62, 93]. In the DINE-CD trial discussed above, the Mediterranean diet performed as well as SCD in producing symptom remission in CD (about 43% at 6 weeks) [77]. While neither diet led to high rates of objective remission in that short time, patients on the Mediterranean diet had comparable improvements in quality of life and symptom scores [77]. Importantly, because the MD is easier to follow and also confers known benefits for cardiovascular health and metabolic parameters, authors suggested it may be the preferred dietary intervention for patients with CD who shows interest in diet-based intervention [77, 94–96]. Moreover, observational studies suggest that greater adherence to a MD pattern is associated with a reduced risk of developing IBD and with a milder disease course [97]. For example, an index-based study found that a high Mediterranean diet score was associated with reduced risk of CD [97]. In UC, evidence is scant – one small study in patients with UC demonstrated improvements in inflammatory markers on a Mediterranean diet, but robust trials are lacking [98]. Mediterranean diet could be safely recommended to patients with IBD due to its anti-inflammatory potential and overall health benefits, although more specific IBD outcomes data were need [99]. In clinical practice, a Mediterranean-style diet is often recommended by gastroenterologists to patients with IBD for its general health benefits [99]. As a primary therapy for inducing remission, MD alone may not be powerful enough (especially in acute moderate-to-severe IBD, where medication is needed), but as a complementary strategy it appears beneficial.

The MD’s anti-inflammatory effects are well-documented in other chronic diseases and likely apply to IBD as well [100, 101]. Key features include a high content of dietary fiber and polyphenols from fruits, veggies, and whole grains, which promote a diverse and SCFA-producing microbiota [102, 103]. Fiber fermentation yields butyrate and other short-chain fatty acids that nourish colonic epithelial cells and downregulate inflammatory pathways [104]. The MD’s emphasis on omega-3 rich fish and monounsaturated fats (olive oil) provides a favorable fatty acid profile – omega-3 fatty acids can compete with arachidonic acid metabolism to reduce pro-inflammatory eicosanoids, and have been shown to help lower TNFα and IL-6 levels in some contexts [105, 106]. Olive oil is also rich in antioxidant compounds (oleic acid, polyphenols) that reduce oxidative stress and inflammation [107]. Meanwhile, the low intake of red and processed meats in MD means less saturated fat and fewer nitroso-compounds and sulfur that can feed sulfide-producing bacteria implicated in colonic inflammation [108]. The MD is also low in refined sugars and ultra-processed foods, which are known to disrupt the microbiome and increase intestinal permeability [109]. By avoiding those, the MD helps maintain gut barrier integrity. In an IBD-specific context, one study found that patients on a MD for 6 months had reductions in fecal calprotectin and CRP compared to baseline [110]. Microbiome analyses show the MD increases beneficial bacterial diversity and abundance of *Faecalibacterium prausnitzii*, a butyrate-producer strongly associated with IBD remission [111]. Indeed, the DINE-CD trial reported that both MD and SCD led to increased microbiome diversity, but MD uniquely was higher in certain vitamin and mineral intake and had additional metabolic benefits (like lower saturated fat intake) [88]. Overall, the MD likely exerts a mild to moderate anti-inflammatory effect through improvements in microbiota composition (more SCFA-producers, fewer pathogenic bacteria) and direct nutrient impacts on immune regulation (e.g., antioxidants, omega-3s) [112]. While it may not induce remission in severe cases, it creates a bodily environment less prone to inflammation.

### Tolerance and Adherence

The MD is highly palatable, culturally adaptable, and easy to follow long-term. It avoids eliminating major food groups, reducing social and psychological burden. In IBD studies, adherence has been high, with patients appreciating its variety and health benefits [113]. Nutritional adequacy is typically not a concern, though some may need guidance to ensure sufficient calories or gradual fiber introduction. Overall, it offers excellent adherence potential and a favorable safety profile, making it a strong option for maintenance or adjunctive dietary therapy in IBD.

### Impact on Microbiota and Inflammation

As mentioned, the MD favorably modulates the gut microbiome. High fiber intake from diverse plant sources increases microbial richness and the production of SCFAs like butyrate [114]. These SCFAs in turn support anti-inflammatory immune responses in the gut (e.g., promoting regulatory T cells) and enhance mucus and barrier function [52, 104, 115]. Studies show MD leads to higher proportions of *Bifidobacterium* and *Lactobacillus* (beneficial genera) and lower proportions of opportunistic bacteria associated with Western diets [116]. To note, it seems that a Mediterranean pattern was associated with reduced levels of inflammatory markers in IBD, including CRP and IL-17 [97]. In general populations, adhering to MD lowers CRP, IL-6, and improves endothelial function, indicating systemic anti-inflammatory effects [117]. In patients with IBD, the MD’s impact on inflammation is likely indirect but positive: by improving the microbiota and nutrient status, it may help keep baseline inflammation lower [118]. For instance, the abundance of antioxidants (vitamins A, C, E, polyphenols) from fruits/veggies can mitigate oxidative stress in inflamed gut mucosa [119]. There is also evidence that the MD can improve the lipid profile of gut membranes (due to high omega-3 and MUFA intake), potentially affecting the assembly of inflammatory cell membranes and cytokine release [120]. While more IBD-specific research is needed, it is reasonable to say the Mediterranean diet *contributes* to an anti-inflammatory milieu and a healthier microbiome, which can support disease control in combination with medical therapy. Its broad health benefits make it a recommended diet for patients with IBD who have no specific contraindications, however ECCO consensus state no evidence-base for induction, despite it is promising [18].

## Low-FODMAP Diet

The Low-FODMAP diet is a specialized elimination diet targeting fermentable carbohydrates that can trigger gastrointestinal symptoms [103, 121, 122]. “FODMAP” stands for fermentable oligosaccharides, disaccharides, monosaccharides, and polyols – these are short-chain carbs that are poorly absorbed and can be rapidly fermented by gut bacteria, producing gas and drawing water into the bowel [123]. The diet was developed for irritable bowel syndrome (IBS) and involves a short-term restriction of high-FODMAP foods (such as wheat, milk, onions, garlic, beans, certain fruits like apples and pears, sweeteners like sorbitol), followed by a structured reintroduction to identify individual tolerances [121–123]. In IBD, the low-FODMAP diet is not intended to treat inflammation, but rather to manage functional symptoms (bloating, abdominal pain, diarrhea) that can persist even when IBD is in remission [18, 124].

Several studies have shown that a low-FODMAP diet can significantly improve gastrointestinal symptoms in IBD patients, particularly those with quiescent or mildly active disease who have IBS-like symptoms [125, ]. A randomized controlled trial by Cox et al. in the UK evaluated a 4-week low-FODMAP diet vs. a sham diet in 52 patients with quiescent IBD and persistent gut symptoms [125]. The low-FODMAP group had a markedly higher rate of adequate symptom relief (50–60% of patients) compared to the control diet group (16%) [125]. IBS severity scores improved significantly on the low-FODMAP diet. Importantly, inflammatory markers (CRP, calprotectin) and disease activity indices remained stable – indicating that symptom improvement was due to functional changes rather than reduced IBD activity [125]. Bodini et al. found that a 6-week low-FODMAP diet in patients with IBD in remission or with mild activity significantly reduced fecal calprotectin levels and improved quality of life, supporting its safety and potential symptomatic benefit in quiescent disease [126]. A recent meta-analysis concluded that low-FODMAP diet is effective for managing functional IBS-type symptoms in IBD, without adverse effects on disease activity [124]. However, it should be emphasized that the low-FODMAP approach is an adjunct for symptom control; it has not been shown to induce remission of IBD inflammation. In fact, it must be used with caution in patients with active IBD, as calorie or nutrient restriction during a flare-up may be counterproductive. For patients with UC and ongoing bloating or urgency despite endoscopic remission, a low-FODMAP diet can be a valuable tool to improve quality of life [127]. In CD, particularly those with post-surgical IBS or functional issues, it likewise can reduce troublesome GI symptoms [127, 128].

### Mechanisms of Action

The low-FODMAP diet works by reducing the fermentative substrate available to colonic bacteria, thereby decreasing gas production and luminal water content [129]. High-FODMAP foods like lactose, fructans, fructose, and polyols can cause gas and osmotic diarrhea in susceptible individuals [129, 130]. By temporarily restricting these, the diet alleviates distension and rapid transit. The symptom improvement is largely mechanical/functional: less fermentation means less gas and bloating, and likely slower colon transit reduces urgency [127, 131, 132]. There is also some evidence that lowering FODMAP load can reduce intestinal permeability in patients with IBS; whether this holds in IBD isn’t confirmed, but if present it could theoretically reduce immune activation by bacterial products [133]. Additionally, the diet might indirectly reduce histamine or serotonin release in the gut (as postulated in IBS) due to less luminal distension [134]. Crucially, the low-FODMAP diet does not directly target inflammation pathways and is not known to reduce cytokine levels or mucosal immune activity in IBD. It’s a symptomatic management strategy.

### Tolerance and Adherence

The low-FODMAP diet is generally implemented in two phases: a strict elimination phase for 4–6 weeks, then a reintroduction phase to liberalize the diet by identifying which specific FODMAP groups the person tolerates [135]. Most patients can adhere to the elimination phase for the short term, especially if they experience quick symptom relief (often within 1–2 weeks bloating and gas improve) [134, 135]. A dietitian’s guidance is very important, as the list of restricted foods is extensive and not always intuitive (for example, wheat bread, milk, apples, garlic, and honey are all high-FODMAP and temporarily off-limits). Patients often report the diet is challenging initially because many common foods are restricted, requiring cooking at home and careful label reading [136]. Adherence to the low-FODMAP diet is generally good because it’s time-limited and patients expect to reintroduce foods. However, some may struggle with the reintroduction phase, becoming overly restrictive out of fear, which risks nutritional deficiencies—especially in fiber and prebiotics. Side effects are minimal, though reduced fiber can cause constipation unless compensated [134]. Psychologically, identifying personal triggers can be empowering and supports long-term adherence to a personalized, less restrictive version of the diet. Overall, motivated patients often adopt a modified low-FODMAP pattern, avoiding only specific problem foods.

### Impact on Microbiota and Inflammation

Short-term low-FODMAP diets do cause changes in the microbiota, some of which could be considered unfavorable if the diet were maintained long-term. A low-FODMAP regimen tends to reduce the overall bacterial abundance, especially gas-producing species [132]. Studies have shown that short-term low-FODMAP diet can lower levels of *Bifidobacteria* (since many FODMAPs are prebiotics that feed them) [131]. This is a potential downside, as *Bifidobacteria* are generally beneficial. Indeed, Cox et al. found that after 4 weeks, patients on low-FODMAP had slightly lower fecal *Faecalibacterium prausnitzii* (an anti-inflammatory bacterium) compared to controls [137]. Interestingly, overall diversity did not change significantly in that short time, but certain functional pathways in the microbiome were altered [131, 137]. This is why the diet is meant to be liberalized – to restore intake of as many fermentable fibers as are tolerated, thereby nourishing the microbiome. Interestingly, in the RCT by Cox et al., while certain bacteria decreased, the overall alpha diversity of the microbiome did not differ between low-FODMAP and control after 4 weeks [137]. This suggests a fairly broad effect rather than wiping out diversity. Markers of inflammation (CRP, calprotectin) in that study showed no significant change [137], indicating that the low-FODMAP diet did not exacerbate inflammation (a theoretical concern was that reducing fiber might increase inflammation, but that wasn’t observed in the short term). Some studies even suggest slight improvement in calprotectin for patients whose baseline diet was very high in FODMAPs and who had a lot of gas-producing bacteria, though this is not consistent [132]. On balance, the low-FODMAP diet improves symptoms by reducing fermentation but may lower beneficial metabolites like butyrate if followed too strictly. To offset this, probiotics or prebiotics are often added after the elimination phase. While it doesn’t directly reduce IBD inflammation, it enhances comfort and may support adherence to healthier long-term eating, aiding overall disease management.

## Plant-Based and Semi-vegetarian Diets

Plant-based diets focus on high consumption of plant foods (vegetables, fruits, legumes, whole grains, nuts) with minimal or no animal products [138]. A semi-vegetarian diet (SVD) or more commonly known as flexitarian, generally means a vegetarian diet with occasional meat [139]. In the context of IBD, the best-known example is the SVD studied by Chiba et al. in Japan, which was a lacto-ovo-vegetarian diet allowing fish once a week and meat only once every two weeks [140, 141]. However, this study was small (n = 22 in the intervention arm), non-randomized, and geographically/culturally specific, raising concerns about selection bias, baseline lifestyle differences, and limited external validity.

Essentially, plant-based diets are rich in fiber and phytonutrients, and low in red meat and animal fat [138]. Vegan diets (no animal products at all) and vegetarian (allowing dairy/eggs) also fall under this umbrella, although specific data in IBD are limited.

A landmark prospective study by Chiba et al. [140] examined diet as maintenance therapy in CD, comparing 22 patients in remission who followed a SVD to 6 control patients who continued an omnivorous diet [142]. The results over 2 years were striking: only 2 of 16 patients who adhered to the SVD relapsed (i.e., 12.5% relapsed, 87.5% stayed in remission), whereas 4 of 6 patients (67%) in the omnivorous diet group relapsed [142]. In absolute terms, the 2-year sustained remission rates were 92% in the semi-vegetarian group vs 33% in the control group [142]. Even at 1 year, the difference was clear (100% vs 67% remission), suggesting a major benefit of a plant-based diet in preventing CD’s flares, arguably one of the best long-term diet results reported [142]. However, the study was small and non-randomized, so bias (the diet group may have been more health-conscious, etc.) could have contributed. A large cohort study from the Swiss IBD Cohort found that while vegetarian and gluten-free diets were not associated with differences in disease activity or outcomes, patients following these diets had significantly altered gut microbiota and reported higher levels of psychological distress, including anxiety, depression, and post-traumatic stress symptoms [143]. A prospective cohort study from the CCFA Partners found that higher dietary fiber intake was associated with a significantly reduced risk of disease flares in CD, but not in UC, suggesting that traditional recommendations to restrict fiber in IBD—particularly CD’s—may warrant reconsideration [144]. For UC, evidence is not as direct, but plant-based diets could theoretically benefit as well – a “UC Vegetarian Diet” (UCVegan) is being explored in research settings. A case series described three patients with UC who achieved and maintained clinical remission for over two years using a whole-food plant-based diet, highlighting its potential as a nonpharmacologic approach that emphasizes anti-inflammatory, gut-supportive foods [145]. Overall, while RCT-level evidence is lacking, the available data and biologic plausibility support that predominantly plant-based diets may help maintain remission, especially in CD.

Emerging evidence suggests that plant-based and SVD exert multiple anti-inflammatory effects through various mechanisms, thereby representing promising adjunctive strategies in the nutritional management of IBD. They are high in fiber, which increases production of short-chain fatty acids (like butyrate) that have healing effects on the colonic mucosa and can regulate immune responses [146]. These diets are naturally low in red meat, reducing intake of heme iron and sulfur-containing amino acids that can be converted into harmful compounds like hydrogen sulfide – this means fewer emulsifiers and thickeners that have been shown to induce colitis in animal models [147]. By emphasizing whole foods, they also avoid additives such as emulsifiers and thickeners linked to gut inflammation [148]. Moreover, plants are rich in antioxidants (vitamin C, E, polyphenols, flavonoids) which scavenge reactive oxygen species in the inflamed gut [149]. Rich in antioxidants and phytochemicals (e.g., curcumin), they help counter oxidative stress. Additionally, plant-based diets support a healthier body weight and metabolic profile, which may indirectly reduce inflammation. Phytochemicals like curcumin (from turmeric, often used in vegetarian Indian diets) have known anti-inflammatory effects in IBD [150]. The high content of omega-6 fatty acids in typical Western diets (from red meat and certain oils) shifts the inflammatory balance; plant-based diets with more omega-3s and less omega-6 can tip that balance toward anti-inflammatory eicosanoid production [151]. Another aspect: plant-based diets tend to promote a healthy body weight and metabolic profile, which might indirectly benefit IBD since obesity can worsen inflammation via cytokines [152]. In Chiba’s study, those on the semi-vegetarian diet had stable BMI and nutritional status, and likely improved their vitamin and mineral intake through diverse plant foods, supporting mucosal healing [141, 153, 154]. Finally, reduction in animal protein intake may lower *protein fermentation* in the colon (which produces ammonia and other irritants) [155].

Nevertheless, the fact that EEN—a highly processed, animal protein-based formula containing several emulsifiers—remains one of the most effective dietary therapies in CD highlights that benefit cannot be explained solely by reducing animal products or ultra-processed food exposure, and that different diets may achieve improvement through distinct mechanisms [55].

### Tolerance and Adherence

Many patients find a vegetarian or semi-vegetarian diet acceptable, especially if they are not required to be 100% vegan [156]. In Chiba’s study, 73% of patients were able to continue the semi-vegetarian diet over 2 years – which indicates decent adherence for a major lifestyle change [141, 154]. Those who had relapses in that study were mostly among the non-adherent or control group [154]. Legumes and soy products provide protein, and the inclusion of eggs/dairy or occasional fish ensures vitamin B12 and iron needs can be met more easily than strict vegan diets. A semi-vegetarian diet is certainly more feasible long-term than extremely restrictive diets like SCD or EEN, because it still offers a wide array of food choices (hundreds of edible plant foods). Nutritional adequacy needs attention – supplementation of vitamin B12 is recommended for strict vegetarians/vegans; iron and calcium intake should be monitored. With guidance, a plant-based diet can be nutritionally complete and even nutritionally superior (high in fiber, vitamins, low in saturated fat). In summary, adherence to a semi-vegetarian diet in motivated patients with IBD can be fairly high, as evidenced by long-term follow-up. It appears to be one of the more sustainable dietary interventions provided the patient is willing and is supported by a dietitian to ensure balanced nutrition.

### Impact on Microbiota and Inflammation

The semi-vegetarian diet study showed remarkably high remission rates in CD over two years, suggesting strong anti-inflammatory effects likely linked to beneficial shifts in the gut microbiota [153, 154]. While in Chiba’s study microbiome data weren’t detailed, high fiber intake presumably increased SCFA producers and butyrate levels, which reduce pro-inflammatory cytokines (like TNFα, IL-6) and support immune regulation [153, 154, 157, 158]. In the study, CRP levels remained low, and relapses correlated with stopping the diet [154]. Vegetarian diets also reduce bile acids production due to lower fat intake, which may reduce colonic injuries [159]. Though evidence in UC is more limited, plant-based diets may similarly enhance butyrate-producing species (e.g., Roseburia, Eubacterium) and support remission [54, 160]. A recent systematic review in UC noted that diets increasing fruits/veggies and reducing meat might help induce remission in mild UC [150]. So, it’s plausible that plant-based diets help quell inflammation in UC as well, though not as conclusively shown as in CD’s. Caution is needed with high fiber in active CD’s, especially with strictures. Overall, plant-based diets appear to foster an anti-inflammatory gut environment and support long-term disease control in IBD, but evidence remains limited and heterogeneous for its use in IBD SPS:refid::bib18SPS:refid::bib18(18).

## Anti-inflammatory Diet (IBD-AID)

The Inflammatory Bowel Disease Anti-Inflammatory Diet (IBD-AID) is a specific dietary regimen developed at the University of Massachusetts, tailored for patients with IBD [161]. It is somewhat based on the SCD but with modifications to broaden it and add functional foods [77, 161]. The IBD-AID has three core principles: (1) restriction of certain carbohydrates (it limits refined sugars, lactose, and most grains, similar to SCD), (2) inclusion of probiotics and prebiotic foods (e.g., yogurt, kefir, certain fibers), and (3) modification of dietary fat intake (emphasizing omega-3s and olive oil, limiting saturated fats) [161]. The diet is implemented in phases: in flares, more easily digestible foods are used (like pureed soups, cooked vegetables, lean protein, fermented foods), and as patients improve, more fiber and raw foods are introduce [162]. Unlike SCD, the IBD-AID eventually permits some gluten-free grains (such as oats, quinoa, rice) and additional plant foods, making it less rigid long-term [161, 163].

The primary evidence for IBD-AID comes from case series and clinical experience, as no RCT has been published solely on this diet yet [161]. A single retrospective case series in 2014 reviewed 40 patients with IBD who were offered the IBD-AID [161]. In that series, 13 patients chose not to attempt the diet, but among the 27 who did, 24 patients (60% of the original 40) achieved a “good” or “very good” clinical response after adhering to the diet, and an additional 3 patients had partial response [161]. In a detailed subset of 11 patients (8 CD, 3 UC), all improved within 4 weeks, with CD’s HBI dropping from 11 to 1.5 and UC Mayo scores to 0, indicating marked improvement [161]. These are encouraging outcomes, albeit uncontrolled. Another small study examined an SCD-like diet (comparable to IBD-AID) in patients with UC and found clinical improvement and microbiome changes, lending indirect support [163]. Currently, a randomized trial of IBD-AID versus a control diet is underway, which should provide higher-quality evidence [164]. Until then, IBD-AID is considered a promising integrative approach but not a stand-alone proven therapy. Both patients with CD and UC have used IBD-AID; the case series included both, and it appears applicable to either disease. Pediatric usage has been less reported, but some centers use a modified IBD-AID in adolescents who cannot do EEN or SCD.

### Tolerance and Adherence

The IBD-AID is moderately restrictive at first, but it offers more flexibility over time than SCD. In the case series, about one-third of patients offered the diet never started it (showing an initial barrier), but among those who gave it a try, adherence was reasonably good [161]. Reasons for initial refusal or drop-out include the effort needed for meal prep and the requirement to avoid convenience foods. However, many find IBD-AID easier to follow than SCD because it reintroduces certain grains and expands allowed foods as you stabilize [165]. The phased, flexible nature of IBD-AID supports better long-term adherence by allowing gradual food reintroduction and avoiding the sense of permanent restriction. Starting with easy-to-digest foods minimizes early GI side effects, and gradual fiber increases are typically well tolerated as inflammation subsides. While more restrictive than the MD, IBD-AID is less so than SCD or EEN, and patients can often navigate eating out with simple modifications. Nutritionally, it’s balanced and covers key macronutrients and micronutrients. Importantly, even partial adherence can provide benefit, making it more sustainable in real-world settings.

### Impact on Microbiota and Inflammation

Preliminary findings suggest that IBD-AID may reduce inflammation and improve gut microbial balance. In the 2014 case series, all adherent patients experienced symptom relief and many discontinued medications, implying inflammation was well controlled [161]. Though biomarkers like CRP and calprotectin weren’t consistently reported, reductions were observed in some patients [161]. Microbiome studies showed increased beneficial bacteria (e.g., Bacteroidetes, Lactobacillus) and decreased Proteobacteria, suggesting less dysbiosis [166, 167]. Stool consistency also improved in all compliant patients, indicating better colonic function [161]. On the inflammation front, one should note that all 8 patients with CD in the detailed review were able to stop steroids or immunomodulators while on IBD-AID and still maintain low symptoms [161, 166]. Not everyone responded (some had mixed results or didn’t follow through), which indicates individual variability – possibly due to differences in baseline microbiome or disease phenotype. Reported improvements are encouraging but must be interpreted cautiously due to the absence of randomization, small sample sizes, and potential adherence bias. A randomized controlled trial is underway, but until such data are available, IBD-AID should be viewed as a promising but unproven approach.

#### Other Diets and Interventions

In addition to the major diets above, various other dietary approaches have been explored in IBD with limited evidence:

- Elimination diets: Some patients attempt elimination of specific items (e.g., dairy-free or gluten-free diets) in hopes of reducing symptoms [168, 169]. However, unless the patient has a true lactose intolerance or celiac disease, simply cutting out dairy or gluten has not shown significant benefit for IBD inflammation [168, 169]. For instance, trials eliminating cow’s milk protein or gluten in UC did not show improvements in clinical or histological outcomes [150, 170]. Only those with demonstrable sensitivities (e.g., concurrent celiac in 2–5% of patients with IBD) benefit from such elimination [171].
- Western Diet” reduction: The flip side of the diets we’ve discussed is the idea of just avoiding “junk food” or heavily processed Western diet elements. While common sense, this strategy hasn’t been rigorously tested as a standalone intervention. Nonetheless, it is generally advised that patients with IBD limit ultra-processed foods, high fructose corn syrup beverages, fried/fatty foods, and excessive red meat [172]. These recommendations come from epidemiological links to IBD flares and the known pro-inflammatory effects of such foods (like increasing endotoxemia) [173]. We can consider this a preventive dietary approach rather than a treatment.
- Ketogenic or Paleo diets: Anecdotally, some patients with IBD try low-carb high-fat diets or paleolithic diets. There is virtually no formal research on ketogenic diets in IBD. Given the high fat content, there could be concerns as high animal fat diets can exacerbate colitis in animal models. Paleolithic diets (which exclude grains, legumes, dairy) have overlap with SCD and IBD-AID principles. One small case series suggested a paleo diet improved symptoms in a few patients with UC, but more data are needed.
- Supplements (Vitamin D & Omega-3): Nutritional supplementation is not a “diet” per se, but since they are diet-derived compounds, it’s worth noting evidence. Vitamin D deficiency is common in IBD and correcting it is important [174, 175]. A few trials suggest high-dose vitamin D may help maintain remission: a meta-analysis found fewer relapses in patients with IBD on vitamin D vs placebo over 12 months (RR 0.57) [176]. One RCT in CD’s showed 13% relapse on vit D (1200 IU/day) vs 29% on placebo [177]. These results hint that ensuring adequate vitamin D (target serum 30–50 ng/mL) could have modest protective effects, likely by immune regulation. A large prospective European cohort study found no significant association between prediagnostic serum or dietary vitamin D levels and the subsequent risk of developing CD or UC, suggesting vitamin D deficiency may not play a major causal role in IBD onset [178]. Omega-3 fatty acids (fish oil) showed early promise, but large high-quality studies have been disappointing. A Cochrane review concluded that omega-3 supplementation is probably ineffective for maintaining CD’s remission (two big trials found no benefit over placebo) [179]. Similarly in UC, fish oil hasn’t consistently prevented flares. Nonetheless, omega-3s have some anti-inflammatory action (lowering leukotriene B4), so some clinicians still advise eating oily fish or taking moderate fish oil as an adjunct, recognizing it likely won’t replace standard therapy [179].
- Probiotics and fiber: While not diets by themselves, adding specific probiotics (like *E. coli* Nissle or *Lactobacillus* mixtures) and soluble fiber supplements (psyllium, pectin) has been studied. In UC, *E. coli* Nissle was shown to maintain remission similarly to mesalamine [6, 180]. Psyllium fiber plus mesalamine improved remission rates in UC in another study [181]. These interventions underscore that manipulating gut flora via targeted additions can help, and they can be considered part of a dietary strategy (e.g., a high-fiber diet with added probiotics). Diets like IBD-AID explicitly incorporate this concept.
- CD-TREAT: it is an ordinary-food diet engineered to replicate the nutritional composition and microbiome/metabolomic effects of EEN [182]. In the feasibility trial by *Svolos *et al*.* CD-TREAT induced clinical remission in 80% of children and 60% of adults with active Crohn’s disease after 4 weeks, with parallel reductions in fecal calprotectin [183]. Metabolomic signatures, bile acid profiles, and microbial shifts closely resembled those seen with EEN, demonstrating that similar anti-inflammatory effects may be achievable using whole foods. However, the sample size was extremely small, the study was short-term, and involved highly selected, well-supported participants, limiting generalizability. CD-TREAT remains an innovative and biologically plausible intervention, but larger randomized controlled trials are required before clinical recommendation.

## Long-Term Outcomes and Considerations

### Disease Course and Relapse Rates

Long-term adherence to a beneficial diet appears to correlate with lower relapse rates in IBD [61, 184, 185]. As discussed, the semi-vegetarian diet study showed a reduced 1–2 year relapse rates in CD’s [143, 154]. Similarly, patients who remain on partial enteral nutrition or CDED maintenance have shown prolonged remission in some case series [58]. However, sustaining dietary changes is challenging, and lapses may lead to symptom return. Some diets (like EEN) are intended only for short-term induction; once normal diet resumes, the disease can flare unless another maintenance strategy is in place [20, 21, 46]. This highlights that diet maintenance is key – introducing a diet for a few weeks may not alter the disease trajectory unless those dietary principles are kept up. For UC, the evidence is less clear on long-term diet impact, but emerging interventions like the UC Exclusion Diet (which combines partial enteral nutrition and avoids certain additives, somewhat analogous to CDED for UC) have shown promise in steroid-free remission for refractory cases [186]. Overall, patients who incorporate a healthy, anti-inflammatory diet as part of their lifestyle (alongside needed medications) often report fewer flare-ups and better overall disease control.

### Quality of Life (QoL)

Effective dietary interventions can substantially improve quality of life in IBD patients. Many diets target symptom relief – reducing daily pain, bloating, urgency can allow patients to return to normal activities and reduce anxiety around eating [124, 126, 187]. Even where diets do not replace medication, patients often feel empowered by having a self-management tool, which can improve mental well-being. The MD, for instance, is associated with improved mood and energy levels, likely due to its dense nutrient content and possible influence on the gut-brain axis [188]. On the flip side, extremely restrictive diets can worsen quality of life if they lead to social isolation or stress around food. It’s a balance: a diet that’s slightly less efficacious but much easier to follow (like MD) might be better for QoL than a highly efficacious but unsustainable one. Most studies report good patient satisfaction in diet groups when the diet works – e.g., in CDED trials, parents and kids were happy to have a food-based option and reported improved family quality of life vs those who had to do formula-only therapy [60, 62, 64].

### Nutritional Status

A crucial goal in IBD management is maintaining proper nutrition, given that malnutrition is common in active disease [189]. Diet interventions must ensure adequate protein, calories, and micronutrients. EEN excels at rapidly correcting malnutrition in pediatric CD’s, often leading to weight gain and catch-up growth [20, 26, 46, 49]. Whole-food diets like MD or plant-based can replete micronutrients if carefully planned. However, overly restrictive regimens risk deficiencies – for example, long-term SCD without supplements could cause folate or B-vitamin deficits due to lack of fortified grains [66]. The IBD-AID was explicitly designed to be nutritionally adequate, including lean meats and grains to cover needs [162]. Regular monitoring of weight and lab markers (iron, vitamin D, B12) is advised when patients are on any significant diet change. In pediatric patients, growth and development must be tracked; diets may need to be liberalized or supplemented to ensure children get enough calcium, vitamin D, protein, and overall energy for growth. Fortunately, many of the diets we reviewed, when done properly, improve nutritional intake promoting the consume of nutrient-dense foods.

### Safety

Most dietary interventions are quite safe if done with guidance. The main risks are malnutrition from overly restrictive practices and potential delay of necessary medical therapy if a patient or provider relies on diet alone inappropriately long. It’s important that diet not be seen as a replacement for proven medications in moderate-severe disease without evidence. Instead, diet is best used as adjunct (or primary in specific mild cases like pediatric CD’s induction with EEN). Some patients may overdo certain diets – e.g., staying in the low-FODMAP elimination phase too long could harm microbiome diversity or cause fiber/vitamin shortfalls. Another example: a vegan diet, if unmonitored, can lead to B12 deficiency which could exacerbate anemia in IBD. Reintroduction and personalization are crucial for safety. Thus, healthcare provider support (dietitian involvement) is recommended to ensure safety.

In conclusion of results, multiple diets have shown ability to induce or maintain remission and/or improve symptoms in IBD to varying degrees. The strength of evidence is highest for EEN (especially in pediatric CD’s) and for CDED + partial EN (recent high-quality trials), moderate for MD and low-FODMAP (for symptom control, supported by RCTs for QOL), and weaker (mostly observational) for SCD, IBD-AID, and plant-based diets—though these latter approaches have many positive anecdotal and physiological rationales. No single diet is a magic bullet, and patient-to-patient variability in response is evident. Accordingly, current evidence supports the adoption of individualized dietary interventions, typically guided by a structured trial-and-error process under clinical supervision, in order to identify a sustainable nutritional strategy that optimally complements pharmacological treatment and ensures adequate nutrient intake.

## Strength of Evidence and Real-World Application of Dietary Strategies in IBD

### Strength of Evidence and Clinical Integration

Based on current evidence, EEN (in pediatric CD’s) stands at the top in terms of proven efficacy (induction of remission comparable to steroids) [19]. It is incorporated into pediatric guidelines as first-line therapy for mild-moderate CD. The CDED, supported by recent RCTs [22], is emerging as a viable alternative to EEN with better adherence – we expect to see it included in future guidelines if results are replicated. The Mediterranean diet and similar whole-food diets have moderate evidence; they may not induce remission alone but are highly sensible for long-term health and possibly to prevent flares [190]. Low-FODMAP diet has strong evidence for symptomatic relief and should be integrated as a management tool for patients in remission who suffer from functional symptoms; gastroenterologists are increasingly referring patients with IBD to low-FODMAP under dietitian supervision to improve quality of life [124, 191]. Diets like SCD and IBD-AID, despite less rigorous evidence, are used by patients; clinicians should be aware of them and provide guidance rather than dismiss them outright, since some patients do report benefit. It may be reasonable to support a trial of SCD/IBD-AID in a motivated patient with mild disease or as adjunct in more severe disease – but with close monitoring and a fallback to medical therapy if there’s no improvement.

### Patient Adherence, Psychosocial Considerations, and Practical Barriers

Adherence remains a central challenge in dietary therapy for IBD. Diets that allow flexibility—such as the Mediterranean, CDED, or semi-vegetarian approaches—tend to see better long-term compliance compared to more rigid regimens like EEN or the SCD. Sustained adherence depends not only on dietary complexity, but also on individual psychosocial and cultural context. For example, children and adolescents often depend on family support for meal preparation and face peer pressures that can hinder strict adherence, especially to restrictive diets [192]. Adults may struggle with social eating or lack motivation when dietary changes feel isolating or overly burdensome. Dietary interventions can empower patients by offering a sense of agency in managing their disease. However, if misapplied or overly restrictive, they may foster disordered eating behaviors, particularly in vulnerable groups. Providers should be attentive to emotional distress, unintentional weight loss, or rigid food rules that signal unhealthy patterns. Cultural preferences and dietary norms should also guide recommendations. Successful implementation often hinges on culturally sensitive adaptations—like identifying familiar low-FODMAP alternatives or plant-based proteins that align with traditional diets. Similarly, financial and practical barriers—such as the cost of specialized products or limited time for meal preparation—can reduce access to therapeutic diets. In such cases, a pragmatic, simplified dietary approach emphasizing reduction of ultra-processed foods and inclusion of whole, nutrient-dense options may offer a more realistic entry point. Ultimately, dietary strategies in IBD should be individualized. Choosing the least restrictive effective option, offering professional support (e.g., dietitians), and framing diet as an adjunct—not a replacement—for medical therapy are key to success in real-world settings.

### Individual Variability in Response

It is evident that not all patients respond to a given diet. Some may flare despite a strict diet, while others achieve remission coincident with dietary change. This variability likely stems from differences in individual disease characteristics (location, behavior of IBD), genetics, baseline microbiome, and perhaps undiscovered food intolerances [193]. For instance, a patient with ileal CD’s might respond well to EEN but a patient with primarily colonic disease might not see as much benefit from diet alone. Likewise, a UC patient with significant dysbiosis might improve on a plant-based diet, whereas another with a more immunologically driven inflammation might need immunosuppression regardless of diet. This underscores a need for personalized nutrition—diets tailored to the person’s microbiome profile and disease state. Future tools, such as microbiome or metabolomic biomarkers, could help predict which patients will benefit from which diet. In practice today, a trial-and-error approach is often used: if one diet fails or is not tolerated, another approach can be tried. Importantly, patients should be cautioned not to blame themselves if a diet doesn’t help – it may simply be that their disease is too aggressive or that diet addresses only one aspect of their illness.

### Role of Healthcare Provider Support

The importance of multidisciplinary support cannot be overstated. Gastroenterologists, dietitians, and psychologists, working together, dramatically increase the success of dietary interventions [194, 195]. Dietitians translate the diet theory into practical grocery lists and recipes, and can ensure nutritional adequacy. Regular follow-up with a dietitian helps troubleshoot adherence issues and adjust the diet as needed. Mental health professionals can assist patients struggling with the emotional burden of dietary limitations or those whose relationship with food is fraught with anxiety [194]. Engaging family members is also beneficial – if the whole family embraces a diet (when feasible), the patient feels less isolated and the diet becomes part of normal routine. An example is pediatric CD’s: families who all eat the CDED meals together see the child have an easier time compared to when the child is singled out with a separate meal [196].

### Medical Therapy Interplay

Dietary therapy should be seen as complementary to pharmacologic therapy, not necessarily as an “alternative.” The ideal approach in difficult IBD might be *combination therapy* – using diet plus medication to achieve deep remission. There is some evidence that diet can enhance drug effectiveness (for instance, one study suggested that combining partial EN with biologics improved outcomes versus biologics alone) [22]. Conversely, a good diet might allow use of lower medication doses or help patients come off steroids faster [161]. However, it’s crucial for patients and providers to communicate – if a patient is trying a diet, the provider should know so they can monitor closely and ensure that, if the patient is failing the diet approach, medical rescue is provided before disease worsens. As we move forward, integrative protocols may emerge (e.g., using EEN or CDED in initial induction, then transitioning to maintenance biologic with a Mediterranean diet).

## Gaps in Research and Future Directions

Despite growing interest in diet as a therapeutic tool for IBD, the evidence base remains limited. Many popular interventions—such as IBD-AID, vegan or semi-vegetarian diets, and even personalized approaches—lack large randomized controlled trials (RCTs). Challenges like blinding, individual variability, and long-term follow-up hinder the generation of robust data. Yet without high-quality trials, integration of dietary strategies into clinical guidelines remains difficult.

A key research gap involves mechanistic understanding. While plausible theories exist—such as modulation of the microbiota, shifts in metabolite production (e.g., short-chain fatty acids), or enhancement of barrier function—more work is needed to pinpoint which dietary components are responsible for therapeutic effects. This could lead to more targeted interventions, where only specific triggers (e.g., emulsifiers, added sugars) are removed or beneficial elements (e.g., prebiotics, fermented foods) added, reducing the need for broad, restrictive regimens.

Future research should also account for population diversity. Most current data come from North America, Europe, or Japan, limiting generalizability. Cultural dietary patterns, baseline microbiome composition, and disease phenotypes (e.g., ileal vs colonic CD’s, left-sided vs pancolitis UC) likely influence response. Stratified studies and more global representation are essential.

## To Advance the Field, Several Promising Directions Emerge

### Personalized Nutrition

Integrating microbiome and genetic data may allow tailored dietary prescriptions. For instance, baseline microbial profiles could identify patients likely to benefit from high-fiber diets, while genetic markers (e.g., IL-23 receptor variants) may suggest who would benefit from specific nutrients or anti-inflammatory compounds.

### Biomarker-Driven Adjustments

Monitoring markers like fecal calprotectin or microbial metabolites (e.g., butyrate, bile acids) could guide dietary decisions. In the future, home-based kits might allow real-time feedback on the impact of diet on inflammation and microbial health.

### Combination Therapy Trials

Diet should not be seen in isolation. Studies exploring synergy between dietary interventions and pharmacologic therapies (e.g., biologics + CDED) are needed. Combination strategies may optimize mucosal healing, reduce medication doses, or lower the risk of post-surgical recurrence.

### Long-Term and Pediatric Studies

Most dietary research is short-term. Trials exceeding one year, particularly in children, are essential to assess sustained remission, growth, and quality of life. Ongoing trials in CDED and IBD-AID are promising, but broader investigations are required.

### Education and Clinical Guidelines

Evidence-based dietary algorithms tailored to clinical context (e.g., low-FODMAP for IBS-type symptoms, EEN for pediatric CD’s) would help clinicians implement nutrition therapy appropriately. Training programs should include nutrition education for both gastroenterologists and dietitians.

Overall, the role of diet in IBD is evolving from a peripheral consideration to a central pillar of integrative care. Within a comprehensive and well-organized IBD center, interdisciplinary management that includes nutritional strategies may offer significant benefits to patients. [195] As research progresses, the goal is to enable clinicians to offer evidence-based dietary guidance alongside medications, empowering patients with tools to actively support their gut health.

## Conclusion

In conclusion, dietary therapy in IBD has moved from the periphery to a more prominent position, supported by growing scientific evidence. However, its efficacy is clearly diet-specific and phenotype-specific: formula-based therapies such as EEN remain most effective in pediatric CD, exclusion diets like CDED show promise in mild–moderate CD, while whole-food patterns or symptom-directed strategies are more appropriate in selected UC or functional-overlay phenotypes.

Across all dietary approaches, adherence remains the central real-world limiting factor, often determining whether clinical remission or symptomatic benefit can be achieved and sustained. This highlights the need for practical, acceptable, and patient-centered nutritional plans developed in collaboration with dietitians.

Looking ahead, microbiome- and biomarker-guided personalization represents a logical and exciting next step. Emerging evidence suggests that specific microbial signatures, inflammatory markers, and food-response phenotypes may help clinicians match the right diet to the right patient, improving both outcomes and adherence.

By embracing diet as a therapeutic ally—and tailoring it according to disease phenotype, patient preferences, and biological signatures—clinicians can deliver truly personalized nutritional management for IBD. This evolution in care holds promise for better long-term outcomes, enhanced quality of life, and greater patient empowerment.

## Data Availability

No datasets were generated or analyzed during the current study.

## Abbreviations

AID: Anti-inflammatory diet
MD: Mediterranean diet
SCD: Specific carbohydrate diet
EEN: Exclusive enteral nutrition
CDED: CD’s disease exclusion diet
SVD: Semi-vegetarian diet
